# Nitric Oxide Synthase 2 (NOS2) Gene Polymorphisms Association With Risk of Pulmonary Tuberculosis (PTB): A Case‐Control Study

**DOI:** 10.1155/bmri/6656158

**Published:** 2026-02-25

**Authors:** Ebrahim Alijani, Mahboobeh Sabeti Akbar-Abad, Hossein Shahriari, Fatemeh Keykha, Mahdi Majidpour, Michael Faqih, Mohsen Taheri, Mahdi Atabaki

**Affiliations:** ^1^ Clinical Immunology Research Center, Zahedan University of Medical Sciences, Zahedan, Iran, zaums.ac.ir; ^2^ Department of Clinical Biochemistry, School of Medicine, Zahedan University of Medical Sciences, Zahedan, Iran, zaums.ac.ir; ^3^ Cellular and Molecular Research Centre, Birjand University of Medical Sciences, Birjand, Iran, bums.ac.ir; ^4^ Department of Biology, Faculty of Science, University of Sistan and Baluchestan, Zahedan, Iran, usb.ac.ir; ^5^ Student Research Committee, Zahedan University of Medical Sciences, Zahedan, Iran, zaums.ac.ir; ^6^ Genetics of Non-Communicable Disease Research Center, Zahedan University of Medical Sciences, Zahedan, Iran, zaums.ac.ir

**Keywords:** nitric oxide synthase 2 (NOS2), polymorphism, pulmonary tuberculosis (PTB)

## Abstract

**Background:**

Pulmonary tuberculosis (PTB) remains a significant global health challenge, necessitating a deeper understanding of genetic factors influencing susceptibility. This study investigates the association between five specific polymorphisms in the nitric oxide synthase 2 (NOS2) genes (rs7215373, rs2297518, rs2274894, rs1800482, and rs9282799) and the risk of developing PTB.

**Methods:**

Utilizing a case‐control design, we analyzed genetic samples from 150 PTB patients and 150 matched healthy controls. Genotyping was conducted using amplification refractory mutation system‐polymerase chain reaction (ARMS‐PCR) methods. Statistical analyses, including logistic regression and Hardy–Weinberg equilibrium tests, were performed to assess the associations between NOS2 polymorphisms and PTB risk.

**Results:**

Our findings indicate that the rs7215373 and rs2274894 polymorphisms had no significant association with the risk of PTB. rs2297518 in the allelic model significantly reduced the risk by 0.50 against the occurrence of PTB (*p* = 0.041). rs1800482 polymorphism in the Codominant 2 (*p* = 0.041), recessive (*p* = 0.043), and allelic (*p* = 0.007) models reduced the risk of PTB by 0.85, 0.80, and 0.75, respectively. However, our results in examining the rs9282799 polymorphism showed that, contrary to previous results, the Codominant 1 (*p* = 0.009), dominant (*p* = 0.005), overdominant (*p* = 0.012), and allelic (*p* = 0.004) models increased the risk of PTB by 3.80, 3.75, 3.78, and 3.49, respectively.

**Conclusion:**

These results suggest that specific NOS2 gene polymorphisms may play a role in modulating the immune response to *Mycobacterium tuberculosis*, highlighting their potential as biomarkers for PTB risk assessment. Further research is warranted to elucidate the underlying mechanisms and to explore the implications for targeted prevention strategies in high‐risk populations.

## 1. Introduction

Pulmonary tuberculosis (PTB) remains one of the leading infectious diseases globally, posing a significant public health challenge, particularly in developing countries [1, 2]. Despite advances in diagnostics, treatment, and preventive strategies, the incidence of PTB continues to rise, highlighting the need for a deeper understanding of the underlying genetic and immunological factors that contribute to susceptibility to this disease [3–5]. One avenue of research that has gained traction is the role of genetic polymorphisms in modulating immune responses to *Mycobacterium tuberculosis* (*Mtb*), the causative agent of PTB or similar diseases such as chronic hepatitis B and COVID‐19 [6–8]. Among the various genes implicated in the immune response, the nitric oxide synthase 2 (NOS2) gene has emerged as a key player due to its critical role in the production of nitric oxide (NO), a potent antimicrobial and immunomodulatory molecule [9, 10].

The NOS2 gene, located on Chromosome 17 [11], codes for the inducible form of NO synthase, which is expressed in response to proinflammatory cytokines during immune activation [12]. NO has been shown to exert direct antimicrobial effects against a variety of pathogens, including *Mtb*, by inhibiting bacterial replication and promoting macrophage activation [13]. The induction of NOS2 is a cornerstone of the protective Th1‐mediated immune response against *Mtb*. Upon infection and phagocytosis by alveolar macrophages, *Mtb* antigens trigger the production of key cytokines, particularly interferon‐gamma (*IFN-γ*) from activated T lymphocytes and natural killer (NK) cells, in conjunction with priming by tumor necrosis factor‐alpha (*TNF-α*) [14]. This cytokine synergy potently upregulates NOS2 expression, leading to the sustained production of large quantities of NO [15]. The antimicrobial action of NO, and its derivative reactive nitrogen intermediates (RNIs), is multifactorial. It directly inflicts nitrosative and oxidative damage on bacterial DNA, proteins, and lipids, and it indirectly compromises *Mtb*′s survival by inhibiting key respiratory enzymes in the *Bacillus* [16]. Furthermore, NO plays a crucial immunomodulatory role by inducing apoptosis in infected macrophages [17], a process that helps to contain the bacteria and facilitate their cross‐presentation to the adaptive immune system, thereby reinforcing the host′s defensive capabilities.

Given its pivotal role in host defense mechanisms, variations in the NOS2 gene may influence the susceptibility to PTB through altered expression or function of the enzyme, thereby impacting NO production [18]. Given this critical nonredundant role of NOS2, the functional integrity of the gene and its regulatory pathways is paramount for an effective host defense [19]. However, the efficacy of this NO‐mediated antimicrobial pathway exhibits significant interindividual variability, a substantial portion of which is attributed to genetic polymorphisms within the NOS2 gene itself [20]. These common genetic variations, including single‐nucleotide polymorphisms (SNPs) in promoter, enhancer, and coding regions, can profoundly influence the quantity, timing, and location of NO production. For instance, promoter SNPs may alter transcription factor binding sites, affecting the inducibility of the gene, while coding SNPs could impact enzyme stability or specific activity [21]. This genetic variation provides a compelling rationale for investigating NOS2 polymorphisms, as they represent a fundamental biological mechanism that could explain differential susceptibility to TB across populations, beyond what can be accounted for by environmental exposure alone.

This case‐control study focuses on investigating the association between specific polymorphisms within the NOS2 gene, namely, rs7215373, rs2297518, rs2274894, rs1800482, and rs9282799, and the risk of developing PTB (Table 1). These polymorphisms have been selected based on previous research suggesting their potential involvement in modulating immune responses and influencing the pathogenesis of infectious diseases. For instance, variations in the NOS2 gene have been linked to altered NO production levels, which may affect the host′s ability to control *Mtb* infection [22]. The significance of understanding these polymorphisms lies not only in elucidating the genetic predisposition to PTB but also in the potential for developing targeted therapeutic strategies and personalized medicine approaches [23]. By identifying individuals at higher risk due to genetic factors, public health interventions can be tailored to improve surveillance, preventive measures, and treatment outcomes for those affected by or at risk of tuberculosis [24].

**Table 1:** Genetic characteristics and population frequencies of the investigated NOS2 polymorphisms.

rs ID	Chromosome position	Cytoband	Common/minor allele	Function/biological effect	Amino acid change	MAF (1000 genomes)
rs7215373	chr17:27748474	17q11.2	T/C	Intronic	—	T = 0.4205
rs2297518	chr17:27769571	17q11.2	G/A	Missense	S608L	A = 0.1653
rs2274894	chr17:27772145	17q11.2	G/T	Intronic	—	T = 0.2678
rs1800482	chr17:27801483	17q11.2	C/G	2 KB upstream variant	—	G = 0.0184
rs9282799	chr17:27801702	17q11.2	G/A	2 KB upstream variant	—	A = 0.0140

Abbreviations: Chr, chromosome; ID, identifier; MAF, minor allele frequency.

In this study, we aim to explore the genetic landscape of NOS2 polymorphisms in relation to PTB in a well‐defined cohort, employing rigorous statistical analysis to establish clear associations. By integrating genetic data with clinical outcomes, this research intends to contribute to the growing body of literature on the genetic determinants of tuberculosis susceptibility, ultimately paving the way for innovative approaches in TB management and prevention. The findings from this study could provide valuable insights into the complex interplay between genetics and infectious diseases, reinforcing the importance of personalized medicine in the fight against tuberculosis.

## 2. Materials and Methods

### 2.1. Study Design

This study was a case‐control design aimed at investigating the association between specific polymorphisms in the NOS2 gene and the risk of developing PTB. The selected polymorphisms included rs7215373, rs2297518, rs2274894, rs1800482, and rs9282799. The study was conducted from April 2023 to October 2024 at Bu‐Ali Hospital, Zahedan, and it was approved by the Zahedan University of Medical Sciences′ ethics committee (The approval certificate can be found at https://ethics.research.ac.ir/IR.ZAUMS.REC.1395.294.). This study adhered to the Declaration of Helsinki ethical guidelines (The 75^th^ WMA General Assembly, Helsinki, Finland, October 2024, https://www.wma.net/policies-post/wma-declaration-of-helsinki/). Informed consent was obtained from all participants prior to inclusion in the study. This case‐control study was designed and reported in accordance with the Strengthening the Reporting of Genetic Association Studies (STREGA) guidelines [25].

### 2.2. Study Population

Healthy control subjects were recruited from the same geographical region (Sistan and Baluchestan province) as the patients to ensure ethnic and sociodemographic matching, thereby minimizing population stratification bias. To achieve a representative sample of the general population without active TB, a multipronged recruitment strategy was employed. Potential controls were invited through announcements in local community health centers and word‐of‐mouth referrals within the same catchment area served by Bu‐Ali Hospital. Unrelated individuals (nonconsanguineous to the cases and without a family history of TB) who accompanied patients to the hospital for reasons unrelated to respiratory illnesses (e.g., routine visits, minor surgical procedures, or as visitors) were informed about the study. Those expressing interest were screened for eligibility. To avoid potential bias related to occupational exposure or a healthcare setting, employees of Bu‐Ali Hospital or other healthcare facilities were not included in the control group. Patients with confirmed PTB were recruited consecutively at the Bu‐Ali Hospital in Zahedan. The initial identification of suspected TB cases occurred at the primary healthcare level through the national TB surveillance network. Individuals presenting with presumptive TB symptoms (e.g., persistent cough, fever, and weight loss) were identified in local health centers across the Sistan and Baluchestan province. These individuals were then formally referred to Bu‐Ali Hospital, the major regional referral center for TB, for definitive diagnosis, specialized care, and registration in the national TB program. Our study recruited patients from this referral pipeline at the point of their confirmed diagnosis at the hospital. Eligibility for inclusion required the patient to have an existing medical record at the hospital documenting the referral and the diagnostic workup. All consecutive patients meeting the microbiological and clinical confirmation criteria (as detailed in Section 2.2.1) during the study enrollment period were invited to participate. This recruitment strategy ensured that our case group comprised a representative sample of bacteriologically confirmed PTB patients from the catchment population of the province.

#### 2.2.1. Case Group

The case group comprised consecutively recruited individuals with a confirmed diagnosis of PTB. All patients were recruited at the time of diagnosis from the Bu‐Ali Hospital in Zahedan, which serves as a major referral center for TB in the Sistan and Baluchestan province, ensuring a representative patient population. The PTB diagnosis was established according to national and international guidelines using a combination of clinical, radiological, and microbiological criteria. Specifically, diagnosis required the presence of consistent clinical symptoms (e.g., persistent cough > 2 weeks, fever, night sweats, and weight loss) along with radiological findings on chest X‐ray or computed tomography (CT) suggestive of active PTB (Figure 1). Crucially, the diagnosis was confirmed microbiologically by either a positive acid‐fast *Bacillus* (AFB) smear and/or a positive culture for *Mtb* from sputum samples. Patients with exclusively extrapulmonary TB or those unable to provide informed consent were excluded from the study.

Figure 1Typical (a) X‐ray and (b) CT for PTB patients.

**Figure:**
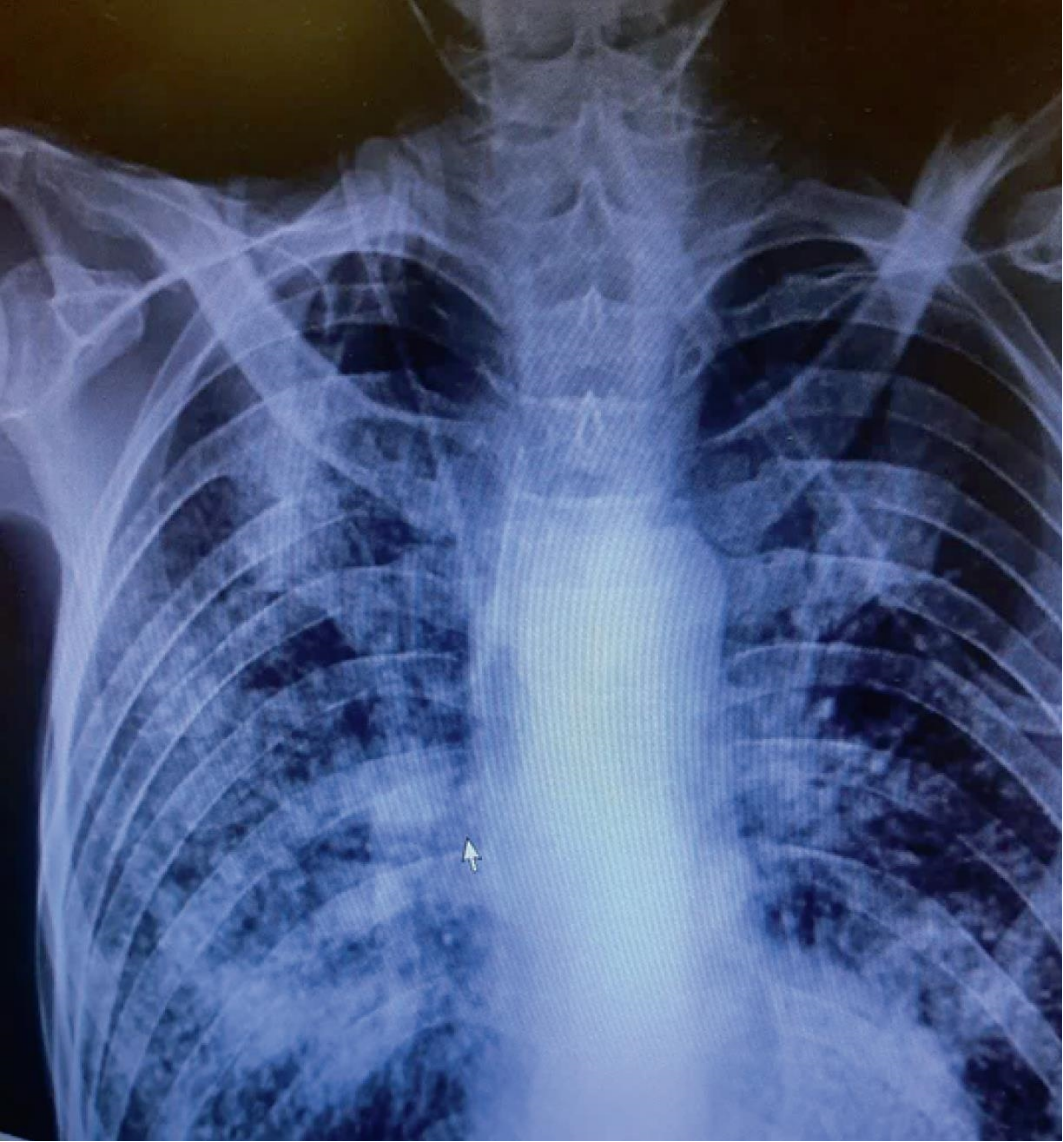
(a)

**Figure:**
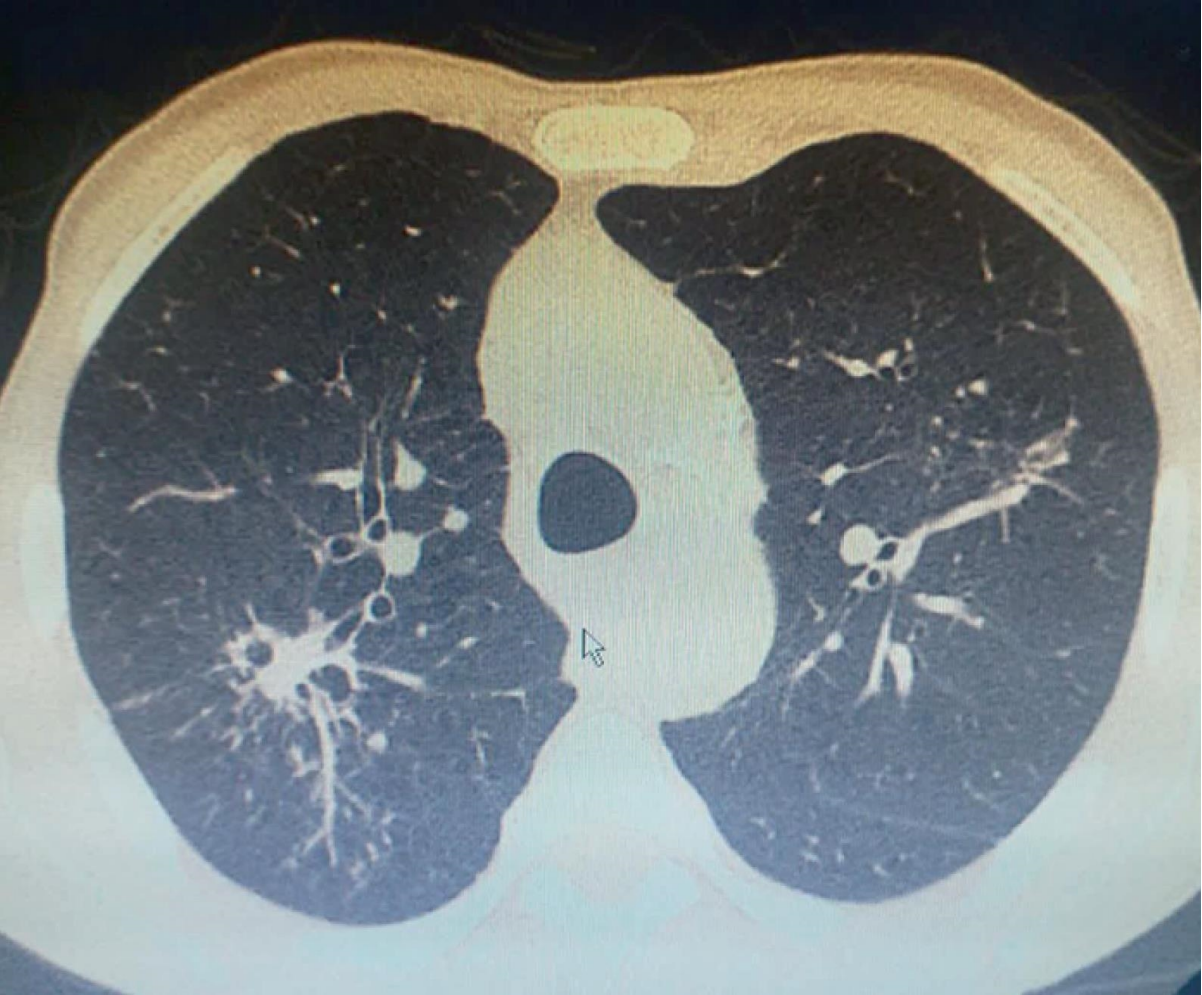
(b)

#### 2.2.2. Control Group

The control group was rigorously selected from the same geographic population as the cases to minimize population stratification bias. All controls were healthy volunteers with no current symptoms or documented history of active tuberculosis. To ensure this, all potential controls underwent a comprehensive screening process that included (1) a detailed medical history questionnaire specifically designed to exclude individuals with any history of TB diagnosis, treatment, or suggestive symptoms (e.g., persistent cough, hemoptysis, night sweats, or unexplained weight loss); (2) the absence of clinical signs of active TB upon brief physical examination; and (3) confirmation of no radiographic evidence suggestive of active or previous TB infection via chest X‐ray review where feasible. Furthermore, individuals with other significant respiratory comorbidities (e.g., asthma, chronic obstructive pulmonary disease, bronchiectasis, or lung cancer) were excluded. Controls were individually matched to cases based on age (±5 years) to control for its potential confounding effect. This meticulous selection process is aimed at creating a control group that accurately represents the genetic background of the source population from which the cases arose, thereby increasing the validity of the observed associations.

#### 2.2.3. Sample Size

A power calculation was conducted to determine the appropriate sample size needed to detect a statistically significant association between NOS2 polymorphisms and PTB risk. A prospective power calculation was performed using G Power to determine the minimum sample size required to detect a significant association between the investigated NOS2 polymorphisms and PTB risk. The calculation was based on the following parameters, derived from prior genetic association studies on NOS2 and TB: a minor allele frequency (MAF) of approximately 0.2, an assumed odds ratio (OR) of 1.8 for disease association, a statistical power of 80% (*β* = 0.20), and a two‐sided significance level of *α* = 0.05. The analysis indicated that a minimum of 142 participants per group (cases and controls) would be required to achieve adequate power. Consequently, we aimed to recruit at least 150 cases and 150 controls for this study. This sample size is consistent with several previously published genetic association studies on tuberculosis and was deemed sufficient to provide reliable results while being feasible within the logistical constraints of our research setting.

### 2.3. Data Collection

#### 2.3.1. Demographic and Clinical Data

Demographic data (age, height, weight, and ethnicity) were collected from all participants using structured questionnaires.

#### 2.3.2. Sample Collection

Peripheral blood samples (2 mL) were obtained from each participant for genetic analysis. Samples were collected in EDTA tubes to prevent coagulation and were stored at −80°C until analysis. Informed consent and ethical approval were confirmed for each participant prior to blood sample collection.

### 2.4. Genetic Analysis

#### 2.4.1. DNA Extraction

Genomic DNA was extracted from peripheral blood leukocytes using the salting‐out method [26]. The quality and concentration of the extracted DNA were assessed (~85 ng/*μ*L) using a NanoDrop spectrophotometer.

#### 2.4.2. SNP Selection

We focused on previously reported SNPs in the NOS2 gene that have been associated with inflammatory responses and susceptibility to infectious diseases. The selected SNPs were rs7215373, rs2297518, rs2274894, rs1800482, and rs9282799 based on a literature review and our previous association findings.

#### 2.4.3. Genotyping

Genotyping of NOS2 SNPs was performed using polymerase chain reaction (PCR) followed by amplification refractory mutation system (ARMS) analysis (Table 2 shows primer sequences used in our study). PCRs were set up in a total volume of 10 *μ*L containing 5 *μ*L Master Mix, 0.9 *μ*L forward primer, 0.9 *μ*L reverse primer, 1 *μ*L template DNA, and 2.2 *μ*L deionized water. The amplification conditions were as follows: initial denaturation at 95°C for 5 min; followed by 34 cycles of 94°C for 30 s, 58°C (rs7215373), 63°C (rs2297518), 59°C (rs2274894), 57°C (rs1800482), and 62°C (rs9282799) for 30 s, and 72°C for 30 s, with a final extension at 72°C for 5 min. Separation of amplified bands was achieved by gel electrophoresis using 2.5% agarose (Figure 2).

**Table 2:** Primer sequences used for genotyping NOS2 polymorphisms.

SNP	Sequence	Genotyping method	Amplified bands (bp)	Annealing temp.
rs7215373 T/C	FO: TGGGCTTTAAGTAATCACCTTGATTGAC	ARMS‐PCR	Outer: 194	58
	RO: AAACAGCAACGTGATGACTCCTCTAGTT		T: 113	
			C: 136	
	R (A): TGCATAAGATGACTAGGCAGCCATTAA			
	F (C): AACACAATCATTTCCTTGTAAGGTGTGAC			

rs2297518 G/A	FO: GGTTTGACCCTCGACACATTC	ARMS‐PCR	Outer: 520	63
	RO: CTCTGGAGAAGGCATGCTACC			
	G/A: 343			

			rs2274894 G/T		FO: CTGGATTCATTGTGAAAGTGTTTCTCA	ARMS‐PCR	Outer: 199	59
RO: GTGCAGGGGAGTCTAGGAAAAGTTAAA								
	G: 152							
	T: 103							
F (G): CATGAAAAACTCATCTACTGTGTAAGTCTG								
R (A): AGGAAAGACTTGTGTGCAGGGATTTCA								

rs1800482 C/G	F (C): CCATGTTGTCCATGCTGCTC	ARMS‐PCR	C/G: 152	57
	F (G): CCATGTTGTCCATGCTGCTG			
	RO: CATATGTATGGGAATACTGTATTTCAG			
rs9282799 G/A	FO: GCCTGAAATACAGTATTCCCATAC	ARMS‐PCR	Outer: 352	62
	RO: GCATTGAGCTGAAGTCTGAAGG			
	F(G): AAGTCACCCTTGATCTCAGCG		G/A: 257	
	F(A): AAGTCACCCTTGATCTCAGCA			

Abbreviations: bp, base pair; F, forward; FO, forward outer; PCR‐RFLP, polymerase chain reaction‐restriction fragment length polymorphism; R, reverse; RE, restriction enzyme; RO, reverse outer; SNP, single‐nucleotide polymorphism.

Figure 2Gel photograph of PCR amplification products of the NOS2 (a) rs7215373 C/T, (b) rs2297518 G/A, (c) rs2274894 G/T, (d) rs1800482 C/G, and (e) rs9282799 G/A polymorphisms.

**Figure:**
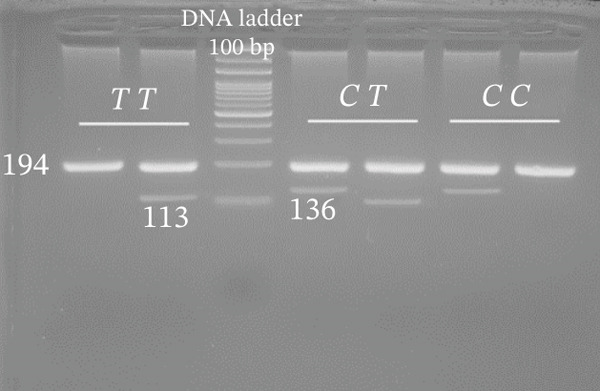
(a)

**Figure:**
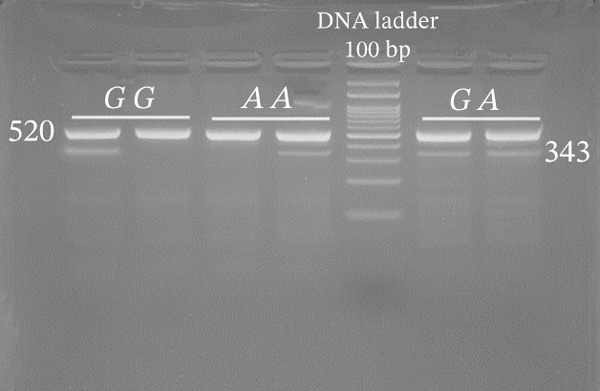
(b)

**Figure:**
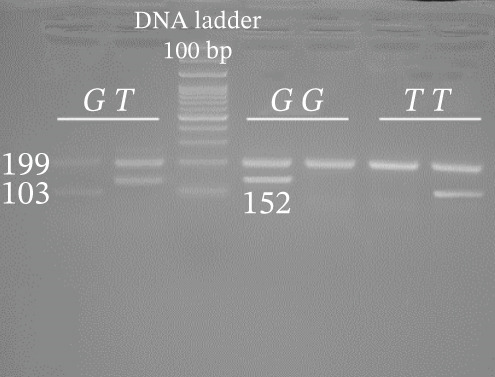
(c)

**Figure:**
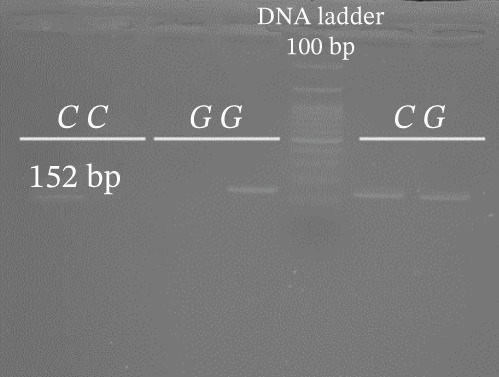
(d)

**Figure:**
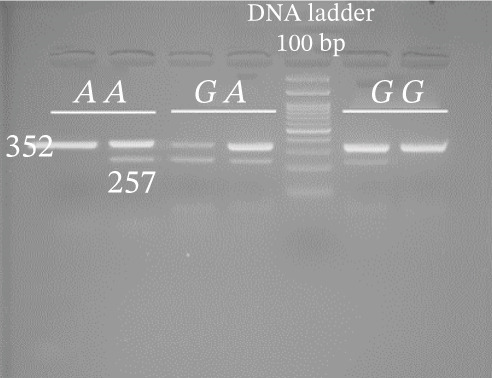
(e)

#### 2.4.4. Quality Control

To ensure the accuracy of genotyping, 10% of the samples were randomly selected for repeat testing. Additionally, negative controls (without DNA) were included in each PCR run to check for contamination.

### 2.5. Statistical Analysis

We used SPSS Statistics Version 27.0.1.0 (IBM Corp., Armonk, New York, United States) for statistical analysis. The genotype frequency distribution was evaluated for Hardy–Weinberg equilibrium with the chi‐square test. Genotypic and allelic frequency disparities between patients and controls were assessed using chi‐square or Fisher′s exact test. ORs and 95% confidence intervals (CIs) were computed by logistic regression analysis and adjusting with ethnicity. A *p* value less than 0.05 was considered statistically significant. To comprehensively assess the association between each polymorphism and disease susceptibility, we evaluated five distinct genetic inheritance models. Codominant models: These models directly compare the genotypes without assuming a specific mode of inheritance. Codominant 1: The heterozygous genotype was compared with the homozygous reference genotype. Codominant 2: The homozygous variant genotype was compared with the homozygous reference genotype. Dominant model: This model tests the effect of the variant allele by comparing the combined heterozygous and homozygous variant genotypes against the homozygous reference genotype. Recessive model: This model tests the effect of the variant allele only in its homozygous state by comparing the homozygous variant genotype against the combined homozygous reference and heterozygous genotypes. Overdominant model: This model specifically tests the effect of the heterozygous state by comparing the heterozygous genotype against the combined homozygous reference and homozygous variant genotypes. Allelic model: This model compares the frequency of the variant allele against the reference allele across all individuals in the case and control groups.

## 3. Results

In this study, we investigated the association between NOS2 polymorphisms and susceptibility to PTB through a well‐defined case‐control approach, encompassing a cohort of 150 individuals diagnosed with PTB and 150 healthy controls.

### 3.1. Demographic Findings

The clinical data of patients and controls are presented in Table 3. The mean age is 38.16 ± 4.68 for controls and 36.19 ± 5.50 in PTB individuals. Age parameters were adjusted for both the case and control groups (*p* = 0.362). The ethnicity of our study population was as follows: In the patient group, there were 64 Fars and 86 Baloch, and in the healthy group, there were 95 Fars and 55 Baloch, and this number was significant (*p* < 0.001) between the two groups. Height (*p* = 0.284) and weight (*p* = 0.104) were not markedly different between the studied groups. The body mass index (BMI) of 108 of the 150 PTB patients ranged from 18.5 to 24.9. So that BMI did not differ significantly in the two groups studied in our study (*p* = 0.230).

**Table 3:** Clinical and demographic characteristics of NOS2 patients and controls.

Parameter evaluated	PTB (*m* *e* *a* *n* ± *S* *D*)	Controls (*m* *e* *a* *n* ± *S* *D*)	*p*
Ethnicity			< 0.001
Fars	64	95	
Baloch	86	55	
Age (year)	36.19 ± 5.50	38.16 ± 4.68	0.362^a^
Height (cm)	170.50 ± 4.68	168.92 ± 3.55	0.284^a^
Weight (kg)	72.18 ± 4.15	75.12 ± 2.10	0.104^a^
BMI (kg/m^2^)	21.74 ± 3.51	22.31 ± 1.30	0.230^a^
Underweight or < 18.5	30	27	
Ideal or 18.5–24.9	108	96	
Overweight or 25–30	11	24	
Obese or > 30	1	3	

Abbreviations: BMI, body mass index; PTB, pulmonary tuberculosis; SD, standard deviation.

^a^Mann–Whitney test. *p* < 0.05 is considered statistically significant.

### 3.2. Genetic Association Analysis

Table 4 displays the genotypic and allelic distribution of NOS2 polymorphisms in the control and PTB cases. Our study revealed that there was no significant association between the rs7215373 C/T and rs2274894 G/T polymorphisms and the risk of PTB. Only in the allelic model (A vs. G), rs2297518 G/A significantly decreased the risk of PTB by 0.50 (OR = 0.50; 95*%*CI = 0.23–0.97; *p* = 0.041). Also, the rs1800482 C/G polymorphism lowered the risk of PTB by 0.85, 0.80, and 0.75 in Codominant 2 (GG vs. CC, OR = 0.15; 95*%*CI = 0.06–1.17; *p* = 0.041), recessive (GG vs. CC + CG, OR = 0.20; 95*%*CI = 0.08–1.16; *p* = 0.043), and allelic (G vs. C, OR = 0.25; 95*%*CI = 0.08–0.69; *p* = 0.007) models. However, our results in examining the rs9282799 G/A polymorphism showed that, contrary to previous results, in Codominant 1 (GA vs. GG, OR = 3.80; 95*%*CI = 1.38–10.61; *p* = 0.009) dominant (GA + AA vs. GG, OR = 3.75; 95*%*CI = 1.47–9.55; *p* = 0.005), over dominant (GA vs. GG + AA, OR = 3.78; 95*%*CI = 1.35–10.37; *p* = 0.012), and allelic (A vs. G, OR = 3.49; 95*%*CI = 1.49–8.25; *p* = 0.004) models increased the risk of PTB by 3.80, 3.75, 3.78, and 3.49, respectively.

**Table 4:** Allelic and genotypic distribution of NOS2 polymorphisms.

SNP	PTB, *n* (%)	Control, *n* (%)	Genetic model	OR (95% CI)∗	*p* ^∗^
rs7215373 C/T					
CC	63 (42.0)	57 (38.0)		1 (reference)	
CT	65 (43.3)	61 (40.7)	Codominant 1	0.98 (0.59–1.61)	0.888
TT	22 (14.7)	32 (21.3)	Codominant 2	0.64 (0.35–1.18)	0.154
HWE	0.438	0.045	Dominant	0.87 (0.56–1.37)	0.484
			Recessive	0.64 (0.36–1.18)	0.135
			Over dominant	1.16 (0.74–1.78)	0.646
C	191 (63.7)	175 (58.3)		1 (reference)	
T	109 (36.3)	125 (41.7)	Allelic	0.85 (0.61–1.16)	0.186
rs2297518 G/A					
GG	141 (94.0)	131 (87.3)		1 (reference)	
GA	7 (4.7)	15 (10.0)	Codominant 1	0.47 (0.18–1.15)	0.079
AA	2 (1.3)	4 (2.7)	Codominant 2	0.52 (0.11–2.62)	0.374
HWE	< 0.001	< 0.001	Dominant	0.45 (0.21–1.09)	0.055
			Recessive	0.49 (0.12–2.76)	0.416
			Overdominant	0.46 (0.19–1.16)	0.084
G	289 (96.3)	277 (92.3)		1 (reference)	
A	11 (3.7)	23 (7.7)	Allelic	0.50 (0.23–0.97)	0.041
rs2274894 G/T					
GG	91 (60.7)	75 (50.0)		1 (reference)	
GT	58 (38.6)	71 (47.3)	Codominant 1	0.68 (0.44–1.10)	0.099
TT	1 (0.7)	4 (2.7)	Codominant 2	0.27 (0.05–1.89)	0.126
HWE	0.011	0.007	Dominant	0.66 (0.48–1.19)	0.070
			Recessive	0.28 (0.08–2.26)	0.185
			Overdominant	0.72 (0.46–1.15)	0.134
G	240 (80.0)	221 (73.7)		1 (reference)	
T	60 (20.0)	79 (26.3)	Allelic	0.78 (0.49–1.05)	0.068
rs1800482 C/G					
CC	147 (98.0)	140 (93.3)		1 (reference)	
CG	2 (1.3)	3 (2.0)	Codominant 1	0.71 (0.15–3.89)	0.624
GG	1 (0.7)	7 (4.7)	Codominant 2	0.15 (0.06–1.17)	0.041
HWE	< 0.001	< 0.001	Dominant	0.29 (0.14–1.12)	0.061
			Recessive	0.20 (0.08–1.16)	0.043
			Overdominant	0.68 (0.13–4.06)	0.655
C	296 (98.7)	283 (94.3)		1 (reference)	
G	4 (1.3)	17 (5.7)	Allelic	0.25 (0.08–0.69)	0.007
rs9282799 G/A					
GG	130 (86.7)	144 (96.0)		1 (reference)	
GA	17 (11.3)	5 (3.3)	Codominant 1	3.80 (1.38–10.61)	0.009
AA	3 (2.0)	1 (0.7)	Codominant 2	3.35 (0.36–32.39)	0.288
HWE	0.014	0.001	Dominant	3.75 (1.47–9.55)	0.005
			Recessive	3.14 (0.32–29.58)	0.320
			Overdominant	3.78 (1.35–10.37)	0.012
G	277 (92.3)	293 (97.7)		1 (reference)	
A	23 (7.7)	7 (2.3)	Allelic	3.49 (1.49–8.25)	0.004

*Note:* Codominant 1 and Codominant 2 represent the heterozygous and homozygous codominant models, respectively. *p* < 0.05 is considered statistically significant.

Abbreviations: CI, confidence interval; HWE, Hardy–Weinberg equilibrium; OR, odds ratio; PTB, pulmonary tuberculosis; SNP, single‐nucleotide polymorphism.

^∗^ *p* value and  ^∗^OR (95% CI), ethnicity adjusted.

## 4. Discussion

PTB remains a significant global health challenge, with millions of new cases reported each year [27]. The disease is caused by the bacterium *Mtb* and is characterized by a complex interplay between host immune responses and pathogen virulence [28, 29]. Among the host factors influencing susceptibility to PTB, genetic variations play a crucial role [30]. This discussion examines the associations of specific NOS2 gene polymorphisms (rs7215373, rs2297518, rs2274894, rs1800482, and rs9282799) with the risk of developing PTB, drawing from a recent case‐control study. NOS2, also known as inducible nitric oxide synthase (iNOS), is an enzyme responsible for the production of NO from L‐arginine [31]. NO is a critical component of the immune response against various pathogens, including *Mtb* [18]. It exhibits antimicrobial properties and plays a role in modulating inflammation [32]. The expression of NOS2 is primarily induced in response to proinflammatory cytokines, such as IFN‐*γ*, which are released during the immune response to infections [33]. The NOS2 gene is located on Chromosome 17 and is subject to various polymorphisms that may influence its expression and activity [11, 34]. These genetic variations can lead to differences in NO production, potentially affecting an individual′s susceptibility to infectious diseases, including PTB [35].

In our case‐control study, we investigated the association between specific NOS2 gene polymorphisms and the risk of PTB. The study involved a cohort of PTB patients and matched healthy controls. The findings revealed significant associations between certain NOS2 polymorphisms and risk of PTB. The identified polymorphisms may influence the transcriptional activity of the NOS2 gene, leading to altered expression levels of the enzyme. For instance, a polymorphism that enhances promoter activity could result in higher NO production, potentially conferring a protective effect against *Mtb*. Conversely, a variant that decreases NOS2 expression may impair the host′s ability to control the infection, increasing susceptibility to PTB. The frequency of NOS2 polymorphisms can vary significantly across different populations due to evolutionary pressures and environmental factors [35]. Our study population may reflect these variations, suggesting that the observed associations might be influenced by the specific genetic background and environmental exposures of the cohort.

NOS2 does not operate in isolation; its activity is closely intertwined with other immune signaling pathways [36]. Variations in the NOS2 gene may interact with polymorphisms in other genes involved in the immune response, such as those coding for cytokines or other NO synthases. Future studies should explore these gene–gene interactions to provide a more comprehensive understanding of the genetic architecture of PTB susceptibility. Understanding the genetic predispositions to PTB can pave the way for personalized medicine approaches. If further validated, genetic testing for NOS2 polymorphisms could be integrated into risk assessment protocols for populations at high risk for tuberculosis. Although our study provides valuable insights, several limitations must be acknowledged. The case‐control design does not establish causality; thus, longitudinal studies are needed to confirm the temporal relationship between NOS2 polymorphisms and PTB development. Additionally, the sample size, although sufficient for statistical analysis, could be expanded to enhance the robustness of the findings and facilitate subgroup analyses. Future research should also focus on functional studies to elucidate the biological mechanisms by which NOS2 polymorphisms influence PTB susceptibility. Investigating the expression levels of NOS2 in different genotypes, as well as the resultant NO production in response to *Mtb* challenge in vitro, could provide critical insights into the interactions between host genetics and pathogen virulence. Lastly, exploring the role of NOS2 polymorphisms in other infectious diseases and inflammatory conditions may help to contextualize the findings within the broader landscape of immunogenetics.

In contrast to the recent study by Pandey et al., which reported a significant association between the T allele of rs2274894 and TB susceptibility (OR = 1.464; 95*%*CI : 1.080–1.983; *p* = 0.014) [37], our analysis did not reveal a significant association for this SNP in our cohort. This discrepancy highlights the potential for population‐specific genetic effects in TB susceptibility. Differences in allele frequency, linkage disequilibrium patterns, or interaction with unique environmental factors in our population may account for the lack of replication. Regarding the rs7215373 polymorphism, our findings diverge from the earlier work of Velez et al. [38], which reported a strong and statistically robust association with TB susceptibility (OR = 1.6;, 95*%*CI = 1.17–2.3;, *p* = 0.004), an association that withstood a false discovery rate correction. In our cohort, however, we observed no significant association for this variant. This discrepancy may be attributed to several factors, including notable differences in the genetic background of the studied populations. The strong linkage disequilibrium patterns present in the population studied by Velez et al. may not be conserved in our population cohort, suggesting that rs7215373 itself might be in linkage with a different, population‐specific causal variant. A compelling contrast emerges from our analysis of the rs2297518 polymorphism. We identified the A allele as a significant protective factor against tuberculosis. This finding directly opposes the results of Alyabyeva et al., who reported the same A allele as a risk factor for a neurovascular overlap syndrome (OR = 8.17; *p* = 0.001). This stark discrepancy strongly suggests that the functional impact of this SNP is disease‐context specific [39]. The NOS2 pathway likely engages in distinct pathophysiological mechanisms in infectious disease (TB) compared with neurovascular disorders, leading to a complete reversal of the allele′s effect. A significant finding of our study was the consistent protective association of the G allele at rs1800482 across Codominant 2, recessive, and allelic genetic models. This stands in contrast to the report by Leandro et al. [40], which found no association between this SNP and TB susceptibility in their Brazilian population subset. The discrepancy suggests that the genetic effect of rs1800482 may be population‐specific and its detection may depend on the application of specific genetic models, underscoring the importance of multimodel analysis in genetic association studies. Our analysis revealed a significant risk association for the A allele of rs9282799 under Codominant 1, dominant, overdominant, and allelic models. This finding stands in contrast to the study by Silva et al. [41], which found no association of this SNP with cervical cancer in a Brazilian population. Notably, Silva et al. acknowledged that their null result may have been due to limited sample size and low statistical power. Our well‐powered study in a TB context provides stronger evidence for the functional relevance of this polymorphism in infectious disease pathogenesis.

Our study reveals a complex and population‐specific landscape of NOS2 genetic associations with tuberculosis. For several polymorphisms, including rs2274894 and rs7215373, our findings diverge from previously reported associations (Pandey et al. [37]; Velez et al. [38]). These discrepancies underscore the substantial role of population genetic structure, where differences in allele frequency, linkage disequilibrium patterns, and gene–environment interactions can profoundly influence the penetrance of genetic risk factors. Furthermore, the protective role of the G allele at rs1800482 in our cohort, undetected in other studies (Leandro et al.), highlights how the application of multimodel genetic analysis is critical for uncovering context‐dependent genetic effects. Strikingly, we identified alleles with opposing effects depending on the disease context. The A allele of rs2297518, which we found to be protective against TB, was reported as a risk factor for a neurovascular disorder (Alyabyeva et al. [39], demonstrating true pleiotropy. Conversely, we established the A allele of rs9282799 as a significant risk factor for TB, a finding strengthened by its contrast with an underpowered study in a different disease (Silva et al. 2022). Collectively, our results move beyond mere replication; they refine the genetic architecture of TB susceptibility by emphasizing population‐specific effects, model‐dependent associations, and the critical influence of pathological context on the functional outcome of NOS2 polymorphisms.

## 5. Conclusion

In conclusion, our case‐control study highlights the association between NOS2 gene polymorphisms and the risk of PTB. The functional implications of these genetic variations underscore the complexity of host–pathogen interactions in PTB. Continued research in this field is essential to unravel the genetic factors contributing to tuberculosis susceptibility, ultimately leading to improved strategies for prevention and treatment. Understanding the genetic underpinnings of PTB can pave the way for personalized medicine approaches, enhancing outcomes for individuals at risk of this pervasive disease.

## Author Contributions

E.A., M.A., and M.T.: methodology and design; M.S.A‐A., M.M., H.S., and F.K.: writing the draft; M.S.A‐A. and M.M.: genotyping; M.S.A‐A. and M.M.: data analysis; H.S.: clinical patient assessment; E.A., M.S.A‐A., M.M., M.A., and M.T.: supervision and editing.

## Funding

The study was funded by the Zahedan University of Medical Sciences (Project No. 7909).

## Ethics Statement

The Ethics Committee at Zahedan University of Medical Sciences approved the study protocol (Ethical Code: IR.ZAUMS.REC.1395.294). A copy of the approval certificate is available at https://ethics.research.ac.ir/IR.ZAUMS.REC.1395.294.

## Consent

Each subject or their legal guardian provided written informed consent.

## Conflicts of Interest

The authors declare no conflicts of interest.

## Data Availability

Access to data will be provided upon reasonable request.
